# Natural Anti-NMDAR1 autoantibodies associate with slowed decline of cognitive functions in Alzheimer’s diseases

**DOI:** 10.1038/s41398-026-03878-x

**Published:** 2026-02-05

**Authors:** Xianjin Zhou

**Affiliations:** 1 Department of Psychiatry, University of California San Diego, La Jolla, CA 92093 USA; 2 VA Research Service, VA Mental Illness Research, Education, and Clinical Center, VA San Diego Healthcare System, San Diego, CA USA

**Keywords:** Psychiatric disorders, Drug discovery

## Abstract

Low titers of blood circulating natural anti-NMDAR1 autoantibodies were reported in ~10% of the general human population. Their potential effects on NMDAR functions in the brain, however, remain unknown. We developed a new method to more accurately quantify these low titers of natural anti-NMDAR1 autoantibodies. After quantifying natural anti-NMDAR1 autoantibodies in the plasma of 324 subjects (163 healthy controls; 161 Alzheimer’s disease (AD) patients), I found that AD patients carrying higher levels of natural anti-NMDAR1 autoantibodies have significantly (*p* value: 0.0015) higher scores of Mini-Mental State Examination (MMSE score: 23.5) than AD patients carrying lower levels of natural anti-NMDAR1 autoantibodies (MMSE score: 21.4). No significant differences in MMSE scores were, however, found between healthy controls with either higher or lower levels of natural anti-NMDAR1 autoantibodies, indicating little harmful effect of the autoantibodies. Consistently, superior cognitive performances were found in AD patients carrying higher levels of natural anti-NMDAR1 autoantibodies in comparison with AD patients carrying lower levels of the autoantibodies. Although this association is intriguing, a causal relationship between natural anti-NMDAR1 autoantibodies and neuroprotection has not yet been established. Since anti-NMDAR1 autoantibodies can bind NMDA receptors to suppress glutamate excitotoxicity in the brain, natural anti-NMDAR1 autoantibodies may have neuroprotective effects against cognitive decline in AD patients.

## Introduction

Alzheimer’s disease (AD) is the most common dementia and affects ~ 5.8 million Americans. AD treatment costed about $305 billion in 2020 and is expected to cost more than $1 trillion as the population ages. Monoclonal antibodies against amyloid-β were recently approved by FDA as disease-modifying therapies for AD with modest and inconsistent effects on slowing down cognitive decline [1, 2]. Potential life-threatening side effects can however occur [3]. More effective and safer treatments to slow down cognitive decline in AD patients are needed.

Different hypotheses for AD development were proposed as reviewed [4]. Glutamate excitotoxicity is one of the potential mechanisms involved in cognitive decline during AD development [5]. In fact, glutamate excitotoxicity is a common mechanism for neuronal injury in both neurological diseases [6] (e.g. stroke, epilepsy) and neurodegenerative diseases [7] (e.g. AD, PD, HD, ALS), and also contributes to the pathogenesis of psychiatric disorders such as schizophrenia [8, 9]. In Alzheimer’s disease, amyloid-β induces glutamate release from both neuronal and glial cells to cause a slow buildup of extracellular glutamate [10, 11] that activates extrasynaptic NMDARs to further increase amyloid-β production [12] and tau over-expression [13], indicating an important role for glutamate excitotoxicity mediated by extrasynaptic NMDARs in exacerbating AD pathogenesis.

Suppression of glutamate excitotoxicity was demonstrated with anti-NMDAR1 autoantibodies in animal models of stroke and epilepsy [14]. Natural anti-NMDAR1 autoantibodies were reported in the blood of ~5–10% of the general human population using semi-quantitative methods [15–18]. It will be interesting to know whether blood natural anti-NMDAR1 autoantibodies may ameliorate glutamate excitotoxicity after crossing blood-brain barriers during AD development [19]. We developed a new method to more accurately quantify blood natural anti-NMDAR1 autoantibodies [20]. Using this new method, I quantified the levels of blood natural anti-NMDAR1 autoantibodies in both healthy controls and early-stage AD patients. I found that AD patients carrying higher levels of natural anti-NMDAR1 autoantibodies have significantly better cognitive performance than AD patients carrying lower levels of natural anti-NMDAR1 autoantibodies.

## Materials and methods

### Plasma samples from healthy controls and AD patients

EDTA plasma samples from Alzheimer’s disease patients (n = 161) and healthy controls (n = 163) were received from Alzheimer’s Disease Research Center (ADRC) at University of California San Diego (UCSD). The original collection of those samples was performed under a protocol approved by the UCSD Human Research Protections Program (IRB Protocol 170957). The plasma samples were selected from the first visit of the subjects at the time of diagnosis of possible AD to avoid complications of medications. More than 50% of possible AD patients were confirmed by pathological studies of postmortem brains. The rest of possible AD patients are still alive. All subjects are over 60 years old; and almost all subjects were examined with Mini-Mental State Examination (MMSE), Clinical Dementia Ranking Sum (CDRSUM), and a subset of subjects received additional cognitive function tests.

### Quantitative immunoassay

Natural anti-NMDAR1 autoantibodies in human plasma samples were quantified using our recently developed method [20]. Goat serum was used as background for negative control. Three human plasma samples with varying high levels of anti-NMDAR1 autoantibodies were used as positive controls and references for normalization between different 96-well plates (Greiner 96-well Flat Bottom Black Polystyrene plate, Cat. No.: 655097). Each human plasma sample was supplemented with the same amount of goat serum as in the negative control for the immunoassay. *Gaussia* luciferase substrate (ThermoFisher, cat. 16160; Pierce™ *Gaussia* Luciferase Glow Assay Kit) was used for quantification on Tecan infinite 200Pro. After subtracting the Relative Light Units (RLU) of the negative control, the remaining RLU would be the levels of natural anti-NMDAR1 autoantibodies in individual plasma samples. A ratio between the RLU of each plasma sample against the average RLU within the same 96-well plate was used as the relative levels of the autoantibodies.

### Statistical analysis

All statistical analyses were performed using R. The effects of naturally occurring anti-NMDAR1 autoantibodies were evaluated on cognitive functions that differ significantly between healthy controls and individuals with Alzheimer’s disease. Tests with more than 10% of randomly missing data were excluded to maintain the validity, reliability, and statistical power of the analyses. Initial evaluation focused on the Mini-Mental State Examination (MMSE), a widely used cognitive test, and the Clinical Dementia Rating Sum of Boxes (CDRSUM), an important functional scale in clinical trials. Additional specific neuropsychological assessments on the autoantibody effects were analyzed thereafter. Neuropsychological outcomes were first examined using analysis of covariance (ANCOVA), incorporating diagnosis and sex as categorical factors and anti-NMDAR1 autoantibody levels as a continuous variable. If no significant sex interaction was detected, data from both sexes were combined for subsequent analyses. Multiple comparison corrections were applied using both false discovery rate (FDR) and permutation-based methods. Outcomes that show significant associations with anti-NMDAR1 autoantibody levels underwent further analysis. Participants in the top quartile of autoantibody levels were categorized into a “high” group, while the remaining individuals will form the “low” group. These neuropsychological outcomes were then re-analyzed using ANOVA, with anti-NMDAR1 autoantibody group, diagnosis, and sex as categorical factors. Tukey’s Honest Significant Difference (HSD) test was used for *post hoc* comparisons. Effect sizes and statistical power were computed to assess the strength and reliability of the findings.

## Results

The total cohort is comprised of 324 subjects, of which 148 (45.7%) are males and 176 (54.3%) are females (Table 1). The cohort is predominantly White (261 [80.6%]), White Hispanic or Latino (51 [15.7%]), and others (12 [3.7%]). There is no age or sex difference between the control and the patient groups. I quantified the levels of natural anti-NMDAR1 autoantibodies in the plasma of 324 subjects using our recently published method [20]. Consistent with our publication [20], specificity of the autoantibody quantification was demonstrated for this cohort (Supplemental Fig. 1). An interaction between sex and diagnosis was found on the levels of natural anti-NMDAR1 autoantibodies (F(1, 320) = 4.15, p = 0.0425) (Supplemental Fig. 2A). *Post hoc* analysis revealed a trend toward elevated levels of natural anti-NMDAR1 autoantibodies in the plasma of male AD patients compared to male controls (Tukey’s HSD, p = 0.055). There is no APOE effect on the autoantibody. However, a significant interaction between Alzheimer’s disease (AD) status and APOE4 genotype was observed on the levels of natural anti-NMDAR1 autoantibodies (F(1,316) = 8.418, p = 0.00398; Supplemental Fig. 2B). *Post hoc* analysis using Tukey’s HSD revealed that AD patients carrying the APOE4 allele exhibited significantly higher levels of natural anti-NMDAR1 autoantibodies compared to AD patients without the APOE4 allele. To investigate the age effect on the levels of the autoantibodies, I separated all subjects into 4 different age groups (Supplemental Fig. 2C). No significant age effect was observed (F(1, 316) = 2.375, p = 0.1243) on the levels of natural anti-NMDAR1 autoantibodies.

**Table 1:** Demographic of the Cohort.

		Total Cohort	AD Patients	Healthy Controls	Test Statistic	*p* value
		(n = 324)	(n = 161)	(n = 163)		
**Age**			74.6 (8.16)	75.9 (5.9)	t test	0.104
**Sex**					x^2^ = 9.3e-05	0.99
					(df=1)	
	Male	148 (45.7%)	73 (45.3%)	75 (46%)		
	Female	176 (54.3%)	88 (54.7%)	88 (54%)		
**Ethnicity**					x^2^ = 2.38	0.304
					(df=2)	
	White	261 (80.6%)	135 (83.9%)	126 (77.3%)		
	White #	51 (15.7%)	22 (13.7%)	29 (17.8%)		
	Others	12 (3.7%)	4 (2.5%)	8 (4.9%)		

Data are mean(SD) or n(%).

# Hispanic or Latino.

**Fig. 1:**
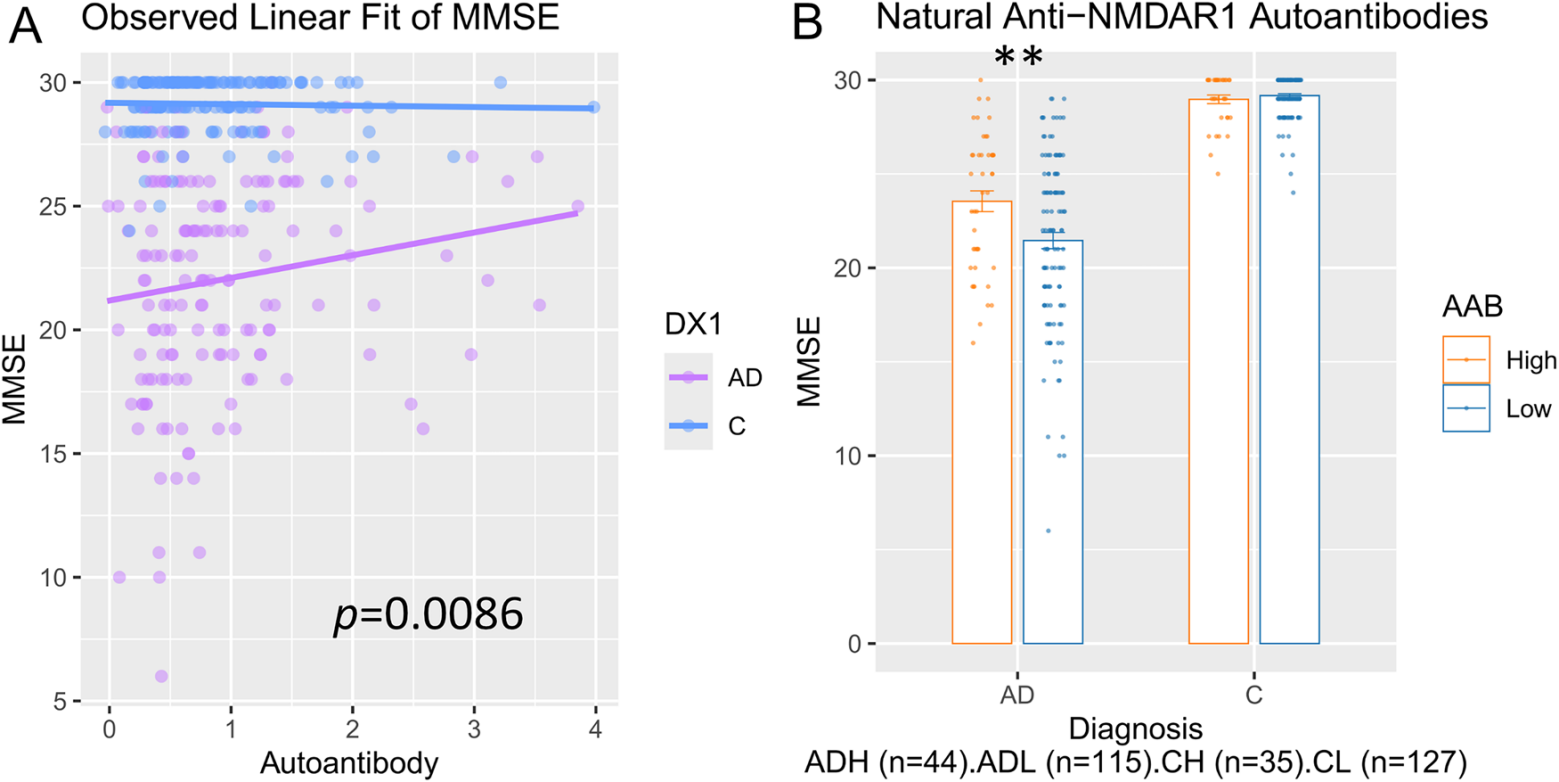
Higher levels of natural anti-NMDAR1 autoantibodies are associated with slowed decline of MMSE scores in AD patients. **A** ANCOVA, using diagnosis as a categorical factor and anti-NMDAR1 autoantibody levels as a continuous variable, revealed a significant association between autoantibody levels and MMSE scores (F(1, 317) = 6.985, *p* = 0.0086). **B** ANOVA analysis, using the autoantibody and diagnosis as categories factors, found a significant interaction between diagnosis and the autoantibody (F(1,317) = 7.597, *p* = 0.00619) on the MMSE scores. *Post hoc* analysis revealed significantly higher MMSE scores in AD patients carrying higher levels of natural anti-NMDAR1 autoantibodies than AD patients carrying lower levels of natural anti-NMDAR1 autoantibodies (Tukey’s HSD, *p* = 0.0015). Data were presented as Mean + SEM. *p* value: ** < 0.01.

The effects of naturally occurring anti-NMDAR1 autoantibodies on the Mini-Mental State Examination (MMSE) and the Clinical Dementia Rating Sum of Boxes (CDRSUM), two widely used cognitive tests in clinical trials, were initially analyzed using analysis of covariance (ANCOVA) with anti-NMDAR1 autoantibody levels as a continuous variable. A significant association was observed between autoantibody levels and MMSE scores (F(1, 317) = 6.985, *p* = 0.0086; Fig. 1A). To further explore the impact of blood natural anti-NMDAR1 autoantibodies on MMSE, subjects were ranked by autoantibody levels and stratified into two categorical groups: the top quartile as the ‘High’ group and the remaining 75% as the ‘Low’ group. No sex effect (F(1, 313) = 0.074, *p* = 0.785) or sex X diagnosis interaction (F(1, 313) = 1.024, *p* = 0.3122) or sex X autoantibody interaction (F(1, 313) = 1.201, *p* = 0.274) were observed on MMSE scores. Therefore, data from both sexes were combined. A significant interaction between diagnosis and the autoantibody (F(1,317) = 7.597, *p* = 0.00619) was observed on the MMSE scores (Fig. 1B). *Post hoc* analysis revealed significantly higher MMSE scores in AD patients carrying higher levels of natural anti-NMDAR1 autoantibodies than in AD patients carrying lower levels of natural anti-NMDAR1 autoantibodies (Tukey’s HSD, *p* = 0.0015). A moderate effect size (Cohen’s d = −0.4767) and decent statistical power (0.7620) were observed. There is no difference in years of education between the two groups (F(1,159) = 0.058, *p* = 0.81). Analysis of covariance (ANCOVA) revealed no significant association between autoantibody levels and CDRSUM scores (*p* = 0.6209; Supplemental Fig. 3A). With the autoantibody as a category factor, there is no autoantibody effect (F(1, 304) = 0.567, *p* = 0.452) or interaction between the autoantibody and diagnosis (F(1, 304) = 0.330, *p* = 0.566) on Clinical Dementia Ranking Sum (CDRSUM)(Supplemental Fig. 3B).

**Fig. 2:**
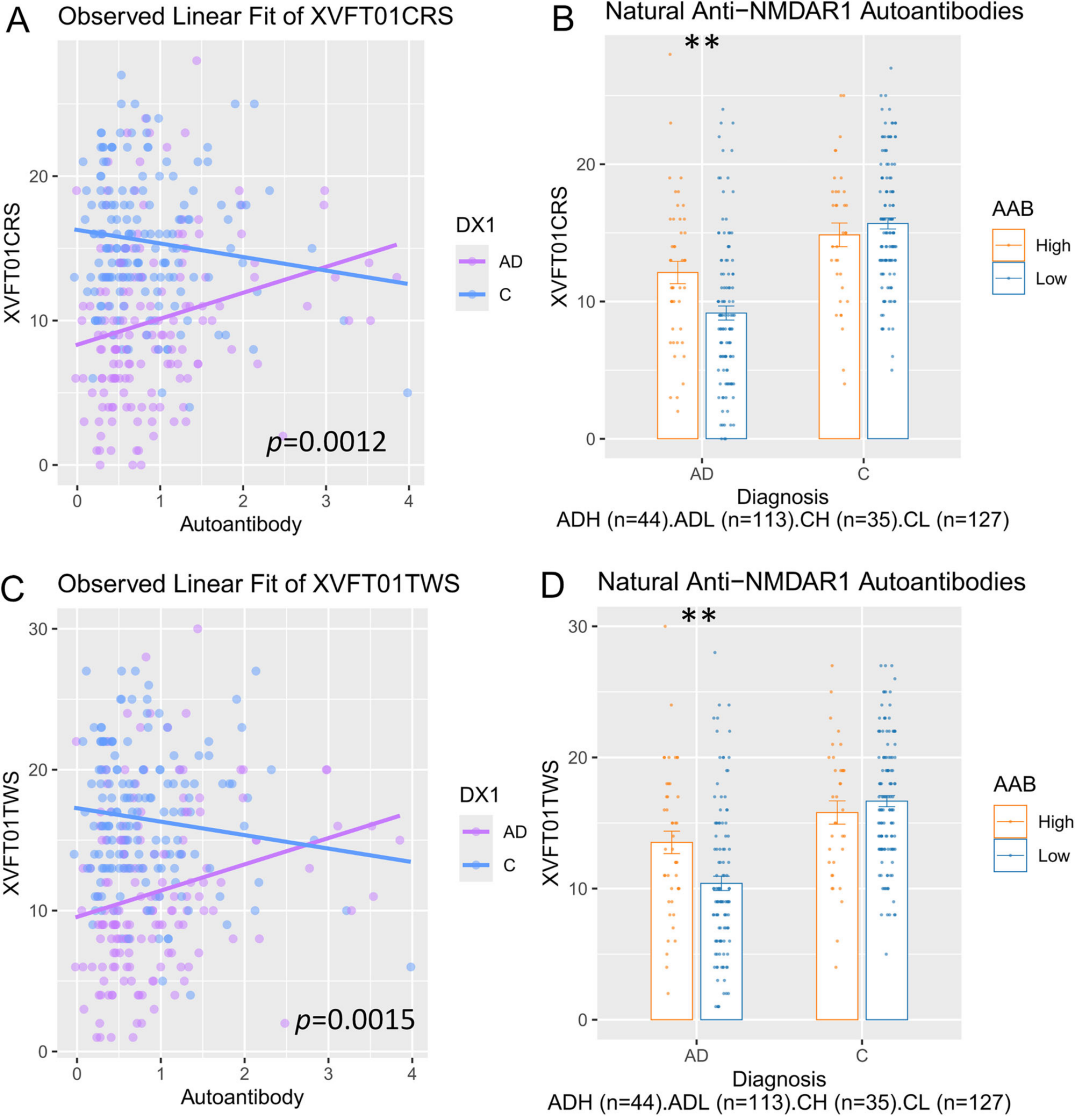
Higher levels of natural anti-NMDAR1 autoantibodies are associated with slowed decline of verbal fluency correct responses and total words in AD patients. **A** ANCOVA, with diagnosis as a categorical factor and anti-NMDAR1 autoantibody levels as a continuous variable, revealed a significant association between autoantibody levels and the number of correct responses in verbal fluency tests using words beginning with ‘S’ (F(1, 315) = 10.682, p = 0.0012). **B** ANOVA using diagnosis and anti-NMDAR1 autoantibody as categorical factors found a significant interaction between diagnosis and the autoantibody with the number of correct responses in verbal fluency tests using words beginning with ‘S’ (F(1, 315) = 8.216, p = 0.0044). *Post hoc* analyses revealed significantly better performances in verbal fluency correct responses using words beginning with ‘S’ (Tukey’s HSD, p = 0.0062) in AD patients carrying higher levels of natural anti-NMDAR1 autoantibodies than AD patients carrying lower levels of natural anti-NMDAR1 autoantibodies. **C** ANCOVA, with diagnosis as a categorical factor and anti-NMDAR1 autoantibody levels as a continuous variable, revealed a significant association between autoantibody levels and the number of total words in verbal fluency tests using words beginning with ‘S’ (F(1, 315) = 10.2747, p = 0.0015). **D** ANOVA using diagnosis and anti-NMDAR1 autoantibody as categorical factors found a significant interaction between diagnosis and the autoantibody with the number of total words in verbal fluency tests using words beginning with ‘S’ (F(1, 315) = 8.22, p = 0.0044). *Post hoc* analysis (Tukey’s HSD, p = 0.0061) revealed significantly better performance in verbal fluency total word production in AD patients with higher levels of natural anti-NMDAR1 autoantibodies than those with lower levels. AD Alzheimer’s Disease, C Healthy Control, F Female, M Male, H High, L Low. Data were presented as Mean + SEM. *p* value: ** < 0.01.

The impact of naturally occurring anti-NMDAR1 autoantibodies on individual cognitive functions—specifically those showing significant differences between healthy controls and individuals with Alzheimer’s disease—was assessed. To ensure analytical validity, reliability, and statistical power, cognitive tests with more than 10% randomly missing data were excluded. ANCOVA analyses, incorporating autoantibody levels as a continuous variable and diagnosis as a categorical factor, with corrections applied using both permutation-based methods and false discovery rate (FDR), are presented in Supplemental Table 1. However, given that these cognitive traits are highly related, multiple comparisons are likely over-corrected. A significant association was found between the autoantibody levels and the number of correct responses in verbal fluency tests using words beginning with ‘S’ (p = 0.0012; Fig. 2A). ANOVA analysis was further conducted with the autoantibody as a category factor. Although sex showed main effects, no significant interactions were detected; therefore, data from both sexes were combined. A significant interaction between AD and autoantibody levels was observed in the number of correct responses on verbal fluency tests using words beginning with ‘S’ (p = 0.0044; Fig. 2B). *Post hoc* analysis (Tukey’s HSD) revealed significantly better performance in Alzheimer’s disease patients with higher levels of natural anti-NMDAR1 autoantibodies compared to those with lower levels (p = 0.0062; Fig. 2B). A moderate effect size (Cohen’s d = −0.5425) and robust statistical power (0.8585) were observed. No difference in years of education exists between the 2 groups. A comparable association was observed in the total word count measures of the verbal fluency tests. ANCOVA analysis with the autoantibody as a continuous variable and diagnosis as a category factor detected a significant association between the autoantibody levels with the total word count measures of the verbal fluency tests using words beginning with ‘S’ (p = 0.0015; Fig. 2C). An additional ANOVA was performed treating autoantibody levels as a categorical variable. Sex showed main effects, but no significant interactions were detected. A significant interaction between Alzheimer’s disease and autoantibody levels was found in total word counts during verbal fluency tests using words beginning with ‘S’ (p = 0.0044; Fig. 2D). *Post hoc* analysis (Tukey’s HSD, p = 0.0061) revealed that Alzheimer’s disease patients with higher levels of natural anti-NMDAR1 autoantibodies performed significantly better than those with lower levels. The autoantibody was associated with a moderate effect size (Cohen’s d = −0.5413) and robust statistical power (0.8571). Effect sizes and statistical power for MMSE and verbal fluency tests are summarized in Table 2.

**Table 2:** Post hoc Analysis, Effect sizes, and Statistical Power.

Test Names	AD-L (n)	AD-H (n)	Tukey’s HSD p_adjusted	Cohen’s d	Statistical Power
**MMSE**	115	44	0.0015	−0.4767	0.7620
**XVFT01CRS**	113	44	0.0062	−0.5425	0.8585
**XVFT01TWS**	113	44	0.0061	−0.5413	0.8571

**Fig. 3:**
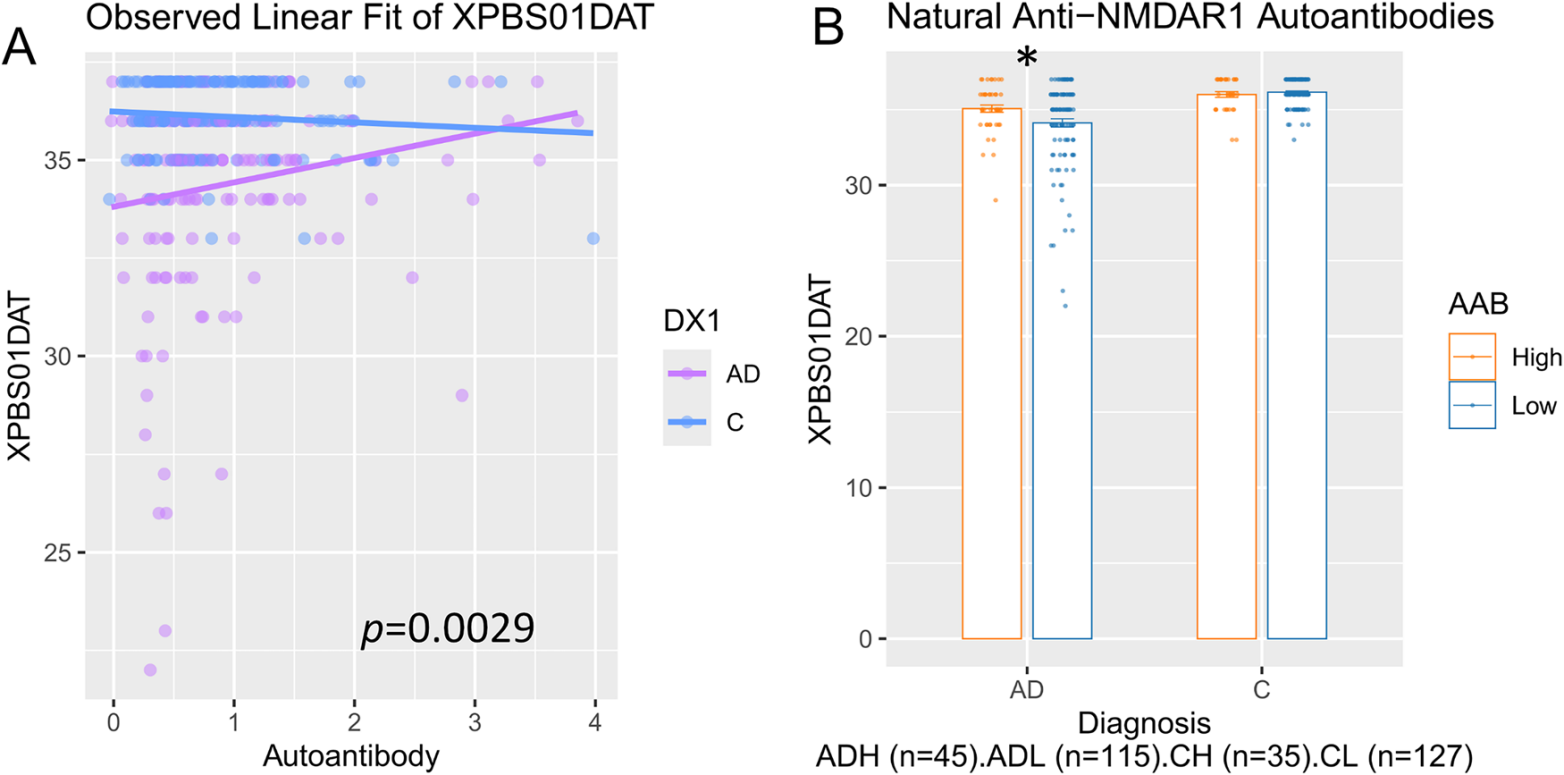
Higher levels of natural anti-NMDAR1 autoantibodies are associated with slowed decline in attention in AD patients. **A** ANCOVA, incorporating diagnosis as a categorical factor and anti-NMDAR1 autoantibody levels as a continuous variable, demonstrated a significant association with attention (F(1, 318) = 8.9781, p = 0.0029). **B** ANOVA using diagnosis and anti-NMDAR1 autoantibody as categorical factors found a significant interaction between diagnosis and the autoantibody on attention (F(1, 318) = 4.718, p = 0.0306). *Post hoc* analyses revealed significantly better performances in attention (Tukey’s HSD, p = 0.032) in AD patients carrying higher levels of natural anti-NMDAR1 autoantibodies. AD Alzheimer’s Disease, C Healthy Control, H High, L Low. Data were presented as Mean + SEM. *p* value: * < 0.05.

In ANCOVA analysis, the autoantibody levels were also associated with attention (p = 0.0029; Fig. 3A). In further analysis with the autoantibody as a categorical factor, a significant interaction was observed between AD and autoantibody levels in attention (p = 0.03, Fig. 3B). *Post hoc* analysis using Tukey’s HSD revealed that AD patients with higher levels of natural anti-NMDAR1 autoantibodies performed significantly better than those with lower levels (p = 0.032). AD patients with higher levels of natural anti-NMDAR1 autoantibodies exhibit better cognitive functions than those with lower levels across all tests listed in Supplemental Table 1, with more representative examples illustrated in Supplemental Figure 4.

## Discussion

I found that higher levels of plasma natural anti-NMDAR1 autoantibodies are associated with significantly slower cognitive decline as measured by MMSE in AD patients. However, no significant difference was found in Clinical Dementia Ranking Sum (CDRSUM). It is possible that more samples are needed to achieve statistical significance in CDRSUM since CDRSUM is more subjective than MMSE. Consistent with higher MMSE scores, AD patients carrying higher levels of natural anti-NMDAR1 autoantibodies have better performance in verbal fluency and attention than AD patients carrying lower levels of natural anti-NMDAR1 autoantibodies. Interestingly, similar findings were reported in schizophrenia where schizophrenia patients carrying natural anti-NMDAR1 autoantibodies display less severity of negative symptoms and better psychosocial function than patients who were negative for the autoantibodies [21, 22]. Given that blood anti-NMDAR1 autoantibodies suppress glutamate excitotoxicity in animal models of stroke and epilepsy [14], it is plausible that plasma natural anti-NMDAR1 autoantibodies may ameliorate glutamate excitotoxicity in brain during AD development. About 0.1% of blood circulating antibodies nonspecifically cross blood-brain barriers into the brain in healthy rodents and humans regardless of antibody specificities or isotypes [23–25]. Blood-brain barriers are compromised in AD patients [26], which may facilitate crossover of plasma anti-NMDAR1 autoantibodies into brain. Since natural anti-NMDAR1 autoantibodies persist for months and years, basal crossover of natural anti-NMDAR1 autoantibodies into brain parenchyma may be sufficient to provide neuroprotective effects via suppressing glutamate excitotoxicity.

Glutamate excitotoxicity is mainly caused by excessive glutamate activating extrasynaptic NMDA receptors (NMDARs) localized outside of the synapse, whereas activation of synaptic NMDARs promotes neuronal survival and mediates synaptic transmission for cognitive functions [27, 28]. Natural autoantibodies in blood are predominantly IgM, with minor contributions from IgA and IgG3 [29]. I hypothesized that IgM anti-NMDAR1 autoantibodies may specifically inhibit extrasynaptic NMDARs to suppress glutamate excitotoxicity, but spare synaptic NMDARs, because IgM antibodies are physically too large to enter the synaptic cleft [19]. Consistent with my hypothesis, memantine that is an NMDAR antagonist preferentially (but not specifically) inhibiting extrasynaptic NMDARs [30] was approved by FDA as a treatment for Alzheimer’s disease. In contrast to IgM, IgG anti-NMDAR1 autoantibodies can access synaptic NMDARs due to their small sizes and thereby may inhibit synaptic NMDA neurotransmission to compromise cognitive functions as shown in patients with anti-NMDAR1 encephalitis [31] and in immunized mice [32].

This study quantified total natural anti-NMDAR1 autoantibodies, which are presumed to be predominantly of the IgM isotype [29]. Quantification of IgM, IgG, and IgA isotype anti-NMDAR1 autoantibodies in AD patients and healthy controls will be needed to differentiate their potential opposite effects on cognitive function in future studies. The potential presence of IgG and IgA natural anti-NMDAR1 autoantibodies in healthy controls may contribute to a slight, non-significant reduction in verbal fluency scores. A larger independent AD cohort will be necessary to replicate the findings and validate the hypothesis. In the future, mice carrying only IgM anti-NMDAR1 autoantibodies could be generated to investigate neuroprotective effects of IgM anti-NMDAR1 autoantibodies. Such experiments would be critical for establishing a causal relationship between natural anti-NMDAR1 autoantibodies and neuroprotection. Demonstrating this causal link may open a new avenue for the development of the IgM antibody therapeutics for AD patients and many other brain diseases and disorders.

## Supplementary information

Supplemental Table 1

Supplemental Figure Legend

Supplemental Figure 1

Supplemental Figure 2

Supplemental Figure 3

Supplemental Figure 4

## Data Availability

The datasets generated and/or analyzed during the current study are available from the corresponding author upon request.
