# Supplementary material for: Natural Anti-NMDAR1 autoantibodies associate with slowed decline of cognitive functions in Alzheimer’s diseases
Source: Transl Psychiatry. 2026 Feb 5;16:92. doi: 10.1038/s41398-026-03878-x (PMC12923874; doi:10.1038/s41398-026-03878-x)
Supplement: Supplementary file 2 — Supplemental Figure Legend [file 41398_2026_3878_MOESM2_ESM.docx]

**Supplemental Figure Legend**

**Supplemental Figure 1. Little contribution from non-specific background binding to high RLU readings of anti-NMDAR1 autoantibodies.** Control wildtype mice (1-4); immunized mice carrying anti-NMDAR1 autoantibodies ^32^ (5-8). Randomly selected human subjects carrying different levels of natural anti-NMDAR1 autoantibodies (9-16). Specificity of the quantification of anti-NMDAR1 autoantibodies were validated in both immunized mice and human subjects using both GLUC and NR1-GLUC probes. The same amount of input GLUC and NR1-GLUC luciferase activities were used for quantification using the same protein AGL mixture to assess both non-specific background binding and specific anti-NMDAR1 antibody binding across mouse and human. There is a very low amount of precipitated GLUC activities across all mouse serum and human plasma samples regardless of the levels of anti-NMDAR1 autoantibodies measured by the NMDAR1-GLUC probe. These data suggested that anti-NMDAR1 autoantibodies were specifically measured for individual plasma samples rather than non-specific bindings from plasma overall general antibodies.

**Supplemental Figure 2. Effects of sex, APOE, and age on the levels of natural anti-NMDAR1 autoantibodies. (A)** An interaction between sex and AD (F(1,320)=4.15, p=0.0425) was found on the levels of natural anti-NMDAR1 autoantibodies. A trend of higher levels of natural anti-NMDAR1 autoantibodies (Tukey’s HSD, p=0.055) in the plasmas of male AD patients than in male healthy controls. **(B)** A significant interaction between AD and APOE4 on the levels of anti-NMDAR1 autoantibody levels (F(1,316) = 8.418, p = 0.00398). *Post hoc* analysis (Tukey’s HSD) revealed that AD patients carrying the APOE4 have significantly higher levels of natural anti-NMDAR1 autoantibodies than AD patients without carrying the APOE4 allele. Subject without (C) or with (P) APOE4. **(C)** All subjects were over 60-years-old and were separated into 4 different age groups (A: 60-69; B: 70-79; C: 80-89; D: >90). No significant age effect was observed (F(1,316)=2.375, p=0.1243) on the levels of natural anti-NMDAR1 autoantibodies between the 4 age groups. Data were presented as Mean+SEM. *p* value: * < 0.05; ^#^ < 0.10.

**Supplemental Figure 3. Effects of natural anti-NMDAR1 autoantibodies on CDRSUM. (A)** There is no significant association between the levels of anti-NMDAR1 autoantibodies and CDRSUM in ANCOVA with diagnosis as a categorical factor and anti-NMDAR1 autoantibody levels as a continuous variate. **(B)** In ANOVA with the autoantibody as a category factor, there is no significant effect on CDRSUM by natural anti-NMDAR1 autoantibodies. AD: Alzheimer’s Disease; C: Healthy Control; H: High; L: Low. Data were presented as Mean+SEM.

**Supplemental Figure 4. AD patients with higher levels of natural anti-NMDAR1 autoantibodies exhibit better cognitive functions than those with lower levels.** ANCOVA, with diagnosis as a categorical factor and anti-NMDAR1 autoantibody levels as a continuous variate, revealed significant associations between autoantibody levels and the number of correct responses in verbal fluency tests using words beginning with 'A' (F(1, 315)=7.3843, p = 0.0069) **(A)** and 'F' (F(1,317)=8.2034, p = 0.0045) **(B)**, and the number of total words in verbal fluency tests using words beginning with 'A' (F(1, 315)=5.9041, p = 0.0156) **(C)** and 'F' (F(1,317)=6.4444, p = 0.0116) **(D)**, as well as a trend toward significance with processing speed and working memory (F(1, 288) = 3.705, p = 0.0552) **(E).**
