# Supplementary figures and images for: Natural Anti-NMDAR1 autoantibodies associate with slowed decline of cognitive functions in Alzheimer’s diseases
Source: Transl Psychiatry. 2026 Feb 5;16:92. doi: 10.1038/s41398-026-03878-x (PMC12923874; doi:10.1038/s41398-026-03878-x)

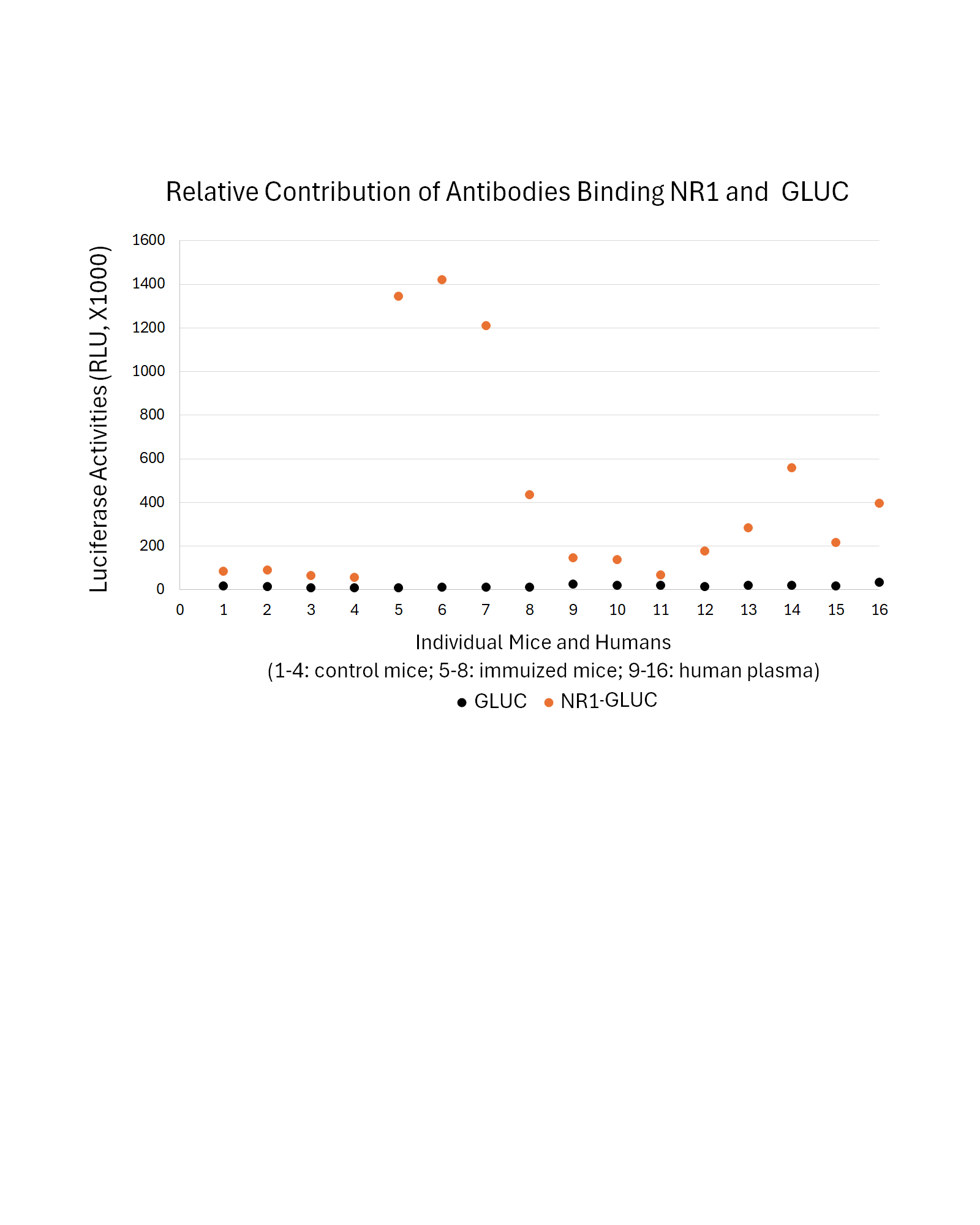

Supplement: Supplementary file 3 — Supplemental Figure 1 [file 41398_2026_3878_MOESM3_ESM.png]

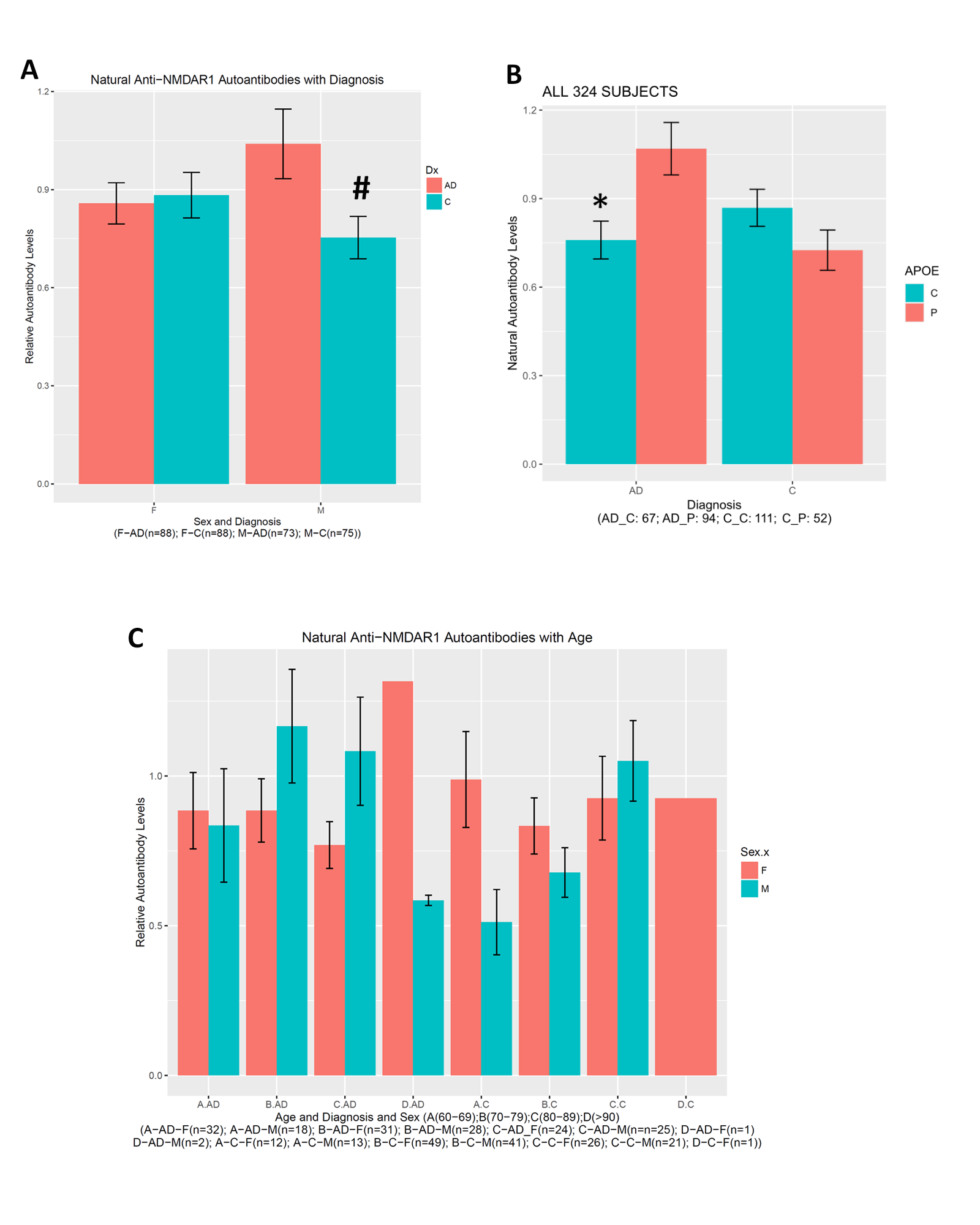

Supplement: Supplementary file 4 — Supplemental Figure 2 [file 41398_2026_3878_MOESM4_ESM.png]

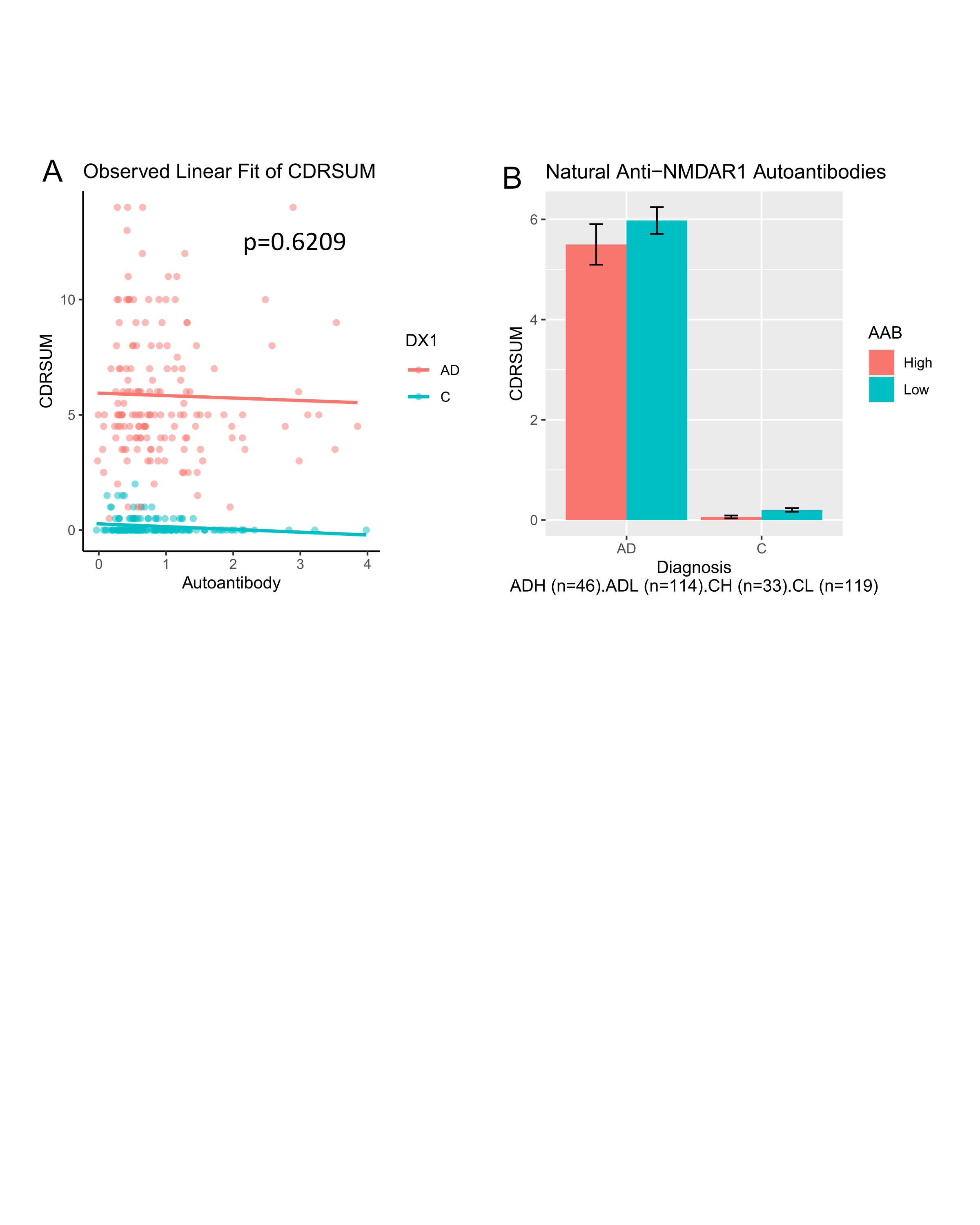

Supplement: Supplementary file 5 — Supplemental Figure 3 [file 41398_2026_3878_MOESM5_ESM.png]

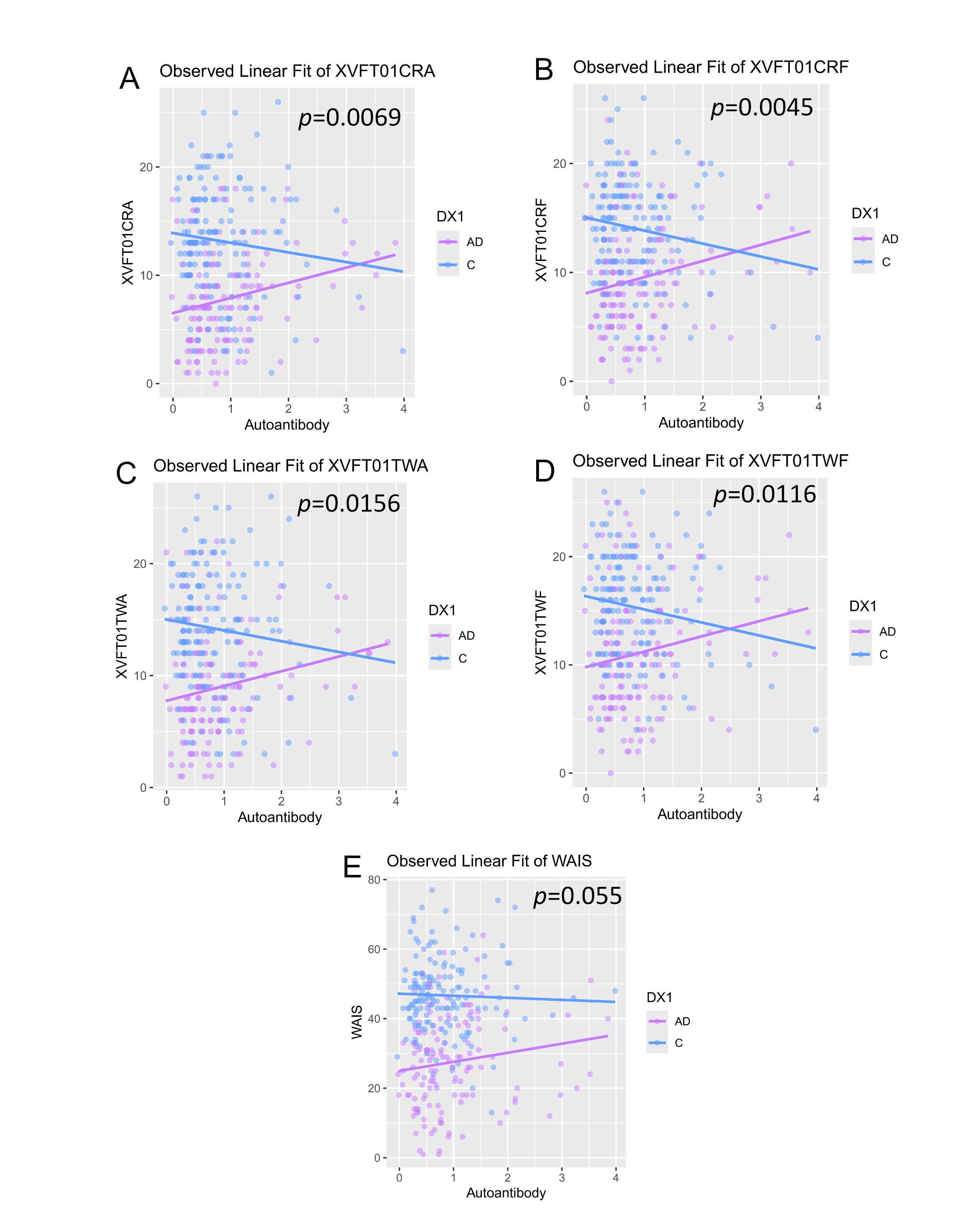

Supplement: Supplementary file 6 — Supplemental Figure 4 [file 41398_2026_3878_MOESM6_ESM.png]
